# Feasibility and Preliminary Efficacy of Empowered Relief in Patients With Chronic Pain Taking Methadone or Buprenorphine: Single-Arm National Pilot Study

**DOI:** 10.2196/86070

**Published:** 2026-03-11

**Authors:** Dokyoung You, Karlyn A Edwards, Maisa S Ziadni, Samsuk Kim, Morgan R Klein, Rachel Cox, Emma Raney, Jay Kuchera, Beth D Darnall

**Affiliations:** 1 The Department of Family and Community Medicine, University of Oklahoma - Tulsa, 4502 E. 41st Street, Tulsa, OK, United States, 1 9186603923; 2 General Internal Medicine, School of Medicine, University of Pittsburgh, Pittsburgh, PA, United States; 3 Division of Pain Medicine, Stanford University School of Medicine, Palo Alto, CA, United States; 4 University of North Carolina, Chapel Hill, NC, United States; 5 Law School, Arizona State University, Phoenix, AZ, United States; 6 Algiatry, Addiction Medicine, Anesthesiology, Port Saint Lucie, FL, United States

**Keywords:** pain management, treatment, behavioral, online, group

## Abstract

**Background:**

Approximately 45% of individuals taking methadone or buprenorphine have chronic pain. These medications are commonly prescribed for chronic pain or opioid use disorder (OUD). To optimize pain management as well as reduce opioid-related symptoms (eg, craving) and risks (misuse and overdose), there is a critical need for a brief, effective, and accessible pain skills intervention for this population.

**Objective:**

This single-arm study aimed to examine the feasibility and preliminary efficacy of online Empowered Relief (ER), a 1-session pain relief skills class, for individuals with chronic pain taking methadone or buprenorphine for chronic pain or OUD.

**Methods:**

A priori feasibility criteria were defined as at least 75% of enrolled participants attending the ER class and the mean satisfaction rating of at least 8 on a 0‐10 scale. Participants were recruited nationally across the United States. Out of the 69 enrolled participants, 55 attended the ER class. Self-report measures were collected at baseline, immediately post class, and at follow-up points of 2 weeks and months 1‐3. Additionally, qualitative interviews were conducted in a small sample (n=14) to obtain in-depth participant feedback. Among the class attendees, 51 participants (27/51, 52.9% female; mean age 48.6, SD 12.2, range 28‐71 years) completed at least one follow-up survey, and treatment outcomes were analyzed using repeated measures ANOVA, with missing data imputed using linear regression. This analytic sample consisted of 24 participants taking methadone and 27 participants taking buprenorphine; 43.1% (22/51) endorsed at least≥2 OUD symptoms within the past 12 months, meeting the *DSM-5* (*Diagnostic and Statistical Manual of Mental Disorders* [Fifth Edition]) diagnostic criteria for current OUD.

**Results:**

Feasibility was achieved with 79.7% (55/74) attendance and mean 8.6 (SD 2) ratings of treatment appraisal and satisfaction. Qualitative feedback demonstrated high acceptability of the class content and delivery, with suggestions for refinements. Repeated-measures ANOVAs and FDR-corrected post hoc tests revealed significant reductions at 1 month post-ER class (primary endpoint) in pain intensity (Cohen *d*=0.71), pain bothersomeness (Cohen *d*=0.54), and pain interference (Cohen *d*=0.61). At 3 months post-ER class, efficacy was maintained for pain intensity, pain bothersomeness, and pain interference (Cohen *d*=0.28, 0.44, and 0.48, respectively). No significant time effects were observed for pain catastrophizing, sleep disturbance, physical function, fatigue, depression, anxiety, social isolation, and opioid craving.

**Conclusions:**

This study is the first to test ER in patients taking methadone or buprenorphine for pain or OUD. Findings showed feasibility, acceptability, and preliminary evidence of treatment efficacy. Participant feedback will inform future study designs. These findings support a randomized trial to fully evaluate the efficacy and scalability of ER in this population.

## Introduction

Chronic pain affects roughly 20% of US adults [1]. Among individuals with chronic pain, nearly one-third report using prescription opioids [2]. However, long-term opioid therapy provides only modest pain relief [3-5] and is often accompanied by adverse side effects [6,7]. Consequently, those taking long-term opioids frequently report insufficient pain relief [7-9], and their overall pain management remains suboptimal. Behavioral pain interventions have emerged as a safe and potentially effective strategy to improve pain-related outcomes, which in turn may help reduce prescription opioid use [10-12]. To date, few studies have examined the effect of behavioral interventions in individuals with chronic pain taking long-term prescription opioids [10,12-14]. While meta-analytic evidence suggests that behavioral pain interventions may lead to moderate reductions in pain intensity and small reductions in opioid use, the overall quality of evidence remains limited [13,14].

Methadone (full mu agonist) [15] and buprenorphine (partial mu agonist) [16] are US Food and Drug Administration–approved and expert-recommended opioid medications for opioid use disorder (OUD) and chronic pain [17,18]. Estimates suggest that 45% of patients taking these medications have chronic pain, regardless of the primary indication for prescription [19,20]. Given that patients taking either of these medications have chronic pain, OUD, or both, targeted behavioral interventions may improve pain outcomes and/or reduce opioid use or misuse. To date, 12-session cognitive behavioral therapy (CBT; 6‐9 h total) and 8-session Mindfulness-Oriented Recovery Enhancement (MORE; 16 h total) are among the few interventions tested in this population. The 12-session CBT for chronic pain has shown no significant effects on pain intensity and pain interference [21], although reductions in opioid misuse have been reported [22]. The 8-session MORE has shown consistent reductions in opioid use and craving across 2 in-person Phase I trials and 1 online Phase III trial [23-25]. However, findings regarding its effects on pain have varied [23-25].

Evidence is promising for behavioral pain treatment in individuals taking medications for OUD; yet, significant barriers impede broad patient access [26-28]. A 1-session pain relief skills intervention (Empowered Relief [ER]) overcomes many existing barriers to care owing to its brief format, and online delivery provides even greater access while keeping treatment burden low [29]. ER has demonstrated efficacy for reducing pain intensity, pain interference, pain bothersomeness, sleep disturbance, depression, and anxiety in heterogeneous pain conditions [29-34]. In-person ER has shown noninferior efficacy compared to traditional, 8-session CBT for chronic low back pain and has produced sustained improvements in pain catastrophizing, pain intensity, pain bothersomeness, and pain interference as well as associated symptoms (eg, fatigue, sleep, depression, and anxiety) up to 3‐6 months [29,30]. When delivered online, ER remains effective in improving pain-related outcomes [31], and has shown preliminary efficacy among individuals with long-term use of short- or long-acting opioids [35]. To date, ER has not been tested in patients with OUD or history of OUD. The current single-arm study sought to test the feasibility and preliminary efficacy of online ER in individuals with chronic pain taking methadone or buprenorphine. We hypothesized that ER would (1) be feasible (as indexed by 75% attendance rate and an average treatment satisfaction rating of ≥8 on a 0‐10 scale) and (2) significantly reduce primary outcomes of interest (pain catastrophizing, pain intensity, pain bothersomeness, and sleep disturbance), improve other outcomes (ie, physical function, fatigue, depression, anxiety, and anger), and reduce opioid craving at 1-month post treatment (primary endpoint). Additionally, we hypothesized that (3) these effects would be maintained at 3 months post treatment (durability endpoint). Finally, we aimed to collect qualitative interview data from a subset of participants to inform potential treatment refinements.

## Methods

### Overview

The Stanford Institutional Review Board approved the study procedures (IRB-60855). This study was preregistered (NCT05057988), and the study protocol is published open access [36]. Informed consent was obtained from all participants. This study was conducted in accordance with the ethical principles outlined in the Declaration of Helsinki. Data were collected using REDCap (Research Electronic Data Capture; Vanderbilt University), a secure, HIPAA (Health Insurance Portability and Accountability Act)-compliant electronic platform. Access to participant data was restricted to a limited number of study personnel, and all data were stored and analyzed on password-protected, encrypted computers.

### Recruitment

**Figure 1.**
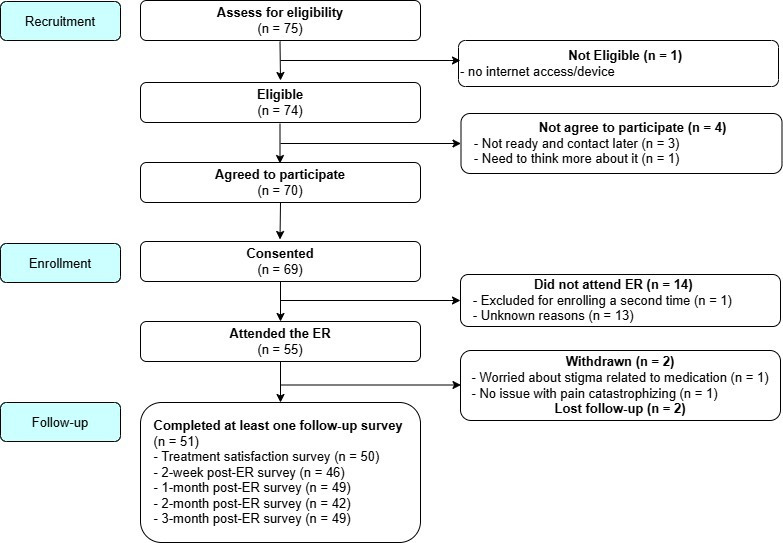
CONSORT diagram. CONSORT: Consolidated Standards of Reporting Trials; ER: Empowered Relief.

Participants were recruited nationally through pain and addiction clinics via physician referrals and self-referral through research posting on a Stanford Pain Division website and the clinicaltrials.gov website. Email invitations were also sent to the Pain Division’s research registry. Study advertisements invited individuals with chronic pain who were taking methadone or buprenorphine to attend a single online pain relief skills class and complete a series of surveys. The study was opened for enrollment in September 2021, and 71 participants were recruited between May 2022 and October 2023 (18 mo). Among the 69 consented participants, 55 attended an ER class (Figure 1).

### Eligibility Criteria

Inclusion criteria were participants >18 years of age, ongoing body pain for more than half of the day for >3 months, currently taking methadone or buprenorphine, and verbal English fluency. To make ER more accessible, internet or computer access was not required. Participants could use any device with video call capability, as long as the referring clinics could accommodate Zoom sessions. Written English fluency was not required as participants had an option of completing their surveys either online, via mailed paper-based format, or with research coordinator-assisted methods. None used the assisted method, and only one participant completed the survey on paper and used pre-paid postal mail to return the survey to the study team. Exclusion criteria were pregnancy or having previously received the ER intervention.

### Procedures

**Figure 2.**
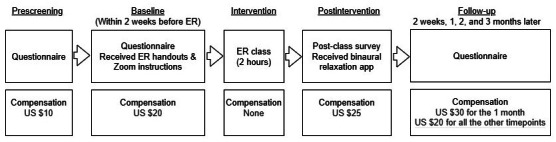
Timeline.

The inclusion and exclusion criteria were posted on the study website and communicated with referring physicians. Individuals who were either self-referred or received the study flyer or study contact information from referring physicians contacted the research coordinator via a phone call, text message, or email. Following initial contact, the research coordinator scheduled a phone call to confirm eligibility (Figure 2) and explain the study goals, procedures, risks, and the compensation schedule. Those expressing interest were sent an electronic consent form and were encouraged to take as much time as needed to review the consent form and contact a research coordinator with any questions.

Following electronic consent, participants received the baseline survey link, zoom instructions, and ER class handouts. During the ER group session, participants were asked to display only their first name and keep their cameras off for privacy. They were encouraged to ask questions either directly to the class instructor or through the online chat function. Upon completion of the ER class, participants received a document of the class slides and binaural app instructions as well as a treatment appraisal and satisfaction survey. Participants received follow-up surveys at 2 weeks, 1 month (primary endpoint), 2 months, and 3 months post treatment (durability endpoint). For the interview portion, we sent an email invitation to the first 20 participants who completed the study, and 14 provided the feedback. Compensation was provided in the form of Amazon gift cards (Figure 2) with amounts ranging from US $10 to US $30, depending on how many surveys a participant completed, and up to $145 possible. Notably, the compensation at 1 month was slightly higher to improve the data collection at the primary endpoint. Those who participated in a 30-minute qualitative interview (n=14) received an additional US $20 Amazon gift card.

### ER

ER is a 2-hour didactic class consisting of pain neuroscience education, experiential and interactive exercises, and 3 core pain management skills such as cognitive reappraisal of pain catastrophizing, self-soothing, and relaxation techniques. During the class, participants self-tailor the content to create a personalized plan for ER, and they receive a binaural calming audio app for downloading onto their smartphone or other devices and with unlimited use. Participants were encouraged to use the calming app daily.

### Data Collection

All study data were obtained via self-report measures and through qualitative interviews (for a subset of participants). The study was designed to be untethered from medical care and as such medical records were not accessed, nor was there any communication with participants’ clinicians.

### Self-Report Measures

Demographic and clinical characteristics were assessed at baseline. The demographics questionnaire included sex, age, race or ethnicity, marital status, education, employment status, household annual income, and housing situation. Clinical questionnaires included pain duration, pain location, pain diagnosis, and mental health diagnoses. Pain duration was assessed using preselected categories, such as (1) <3 months, (2) between 3 and <6 months, (3) between 6 and 12 months, and (4) >1 year. Pain diagnoses, opioid medication, and mental health diagnoses were assessed in a free text format. Additionally, prior experience with a pain self-management class was asked in a yes or no question. Finally, the *DSM-5* (*Diagnostic and Statistical Manual of Mental Disorders* [Fifth Edition]) OUD symptom checklist was administered to evaluate the participants’ current OUD severity (past 12 mo) [37,38].

To assess the primary study outcome of feasibility and acceptability, 11 questions [36] were asked to evaluate (1) overall satisfaction, (2) ease of understanding the class content, (3) relevancy, (4) usefulness, (5) likelihood of using the skills, (6) likelihood of recommending the class to others, (7) ease of operating Zoom, (8) concerns about privacy while attending the Zoom class, (9) feeling comfortable engaging with the Zoom instructor and class participants, (10) feeling of connection with the instructor, and (11) preference for a single, 2-hour class or two 1-hour classes. All questions were rated on a 0‐10 scale except for the last question with 3 response options (1: prefer single, 2-h class; 2: prefer 1-h two classes; and 3: other). When selecting “Other” as a response, a free text box was generated to elicit detailed information. To ensure consistency in directionality of scale items, (8) the concern about privacy was reverse coded. To gain deeper insight into acceptability and future refinements, structured qualitative interviews were conducted with 14 volunteers after attending the class.

Preliminary treatment efficacy was assessed with the following patient-reported outcome measures. First, the 13-item Pain Catastrophizing Scale (PCS) was administered to assess the levels of pain catastrophizing (α=.87) on a 5-point scale from 0 (not at all) to 4 (all the time) [39]. Pain catastrophizing is a maladaptive cognitive-emotional response to pain, characterized by magnification, rumination, and helplessness about pain [39]. The PCS total score ranges from 0 to 52, with higher scores indicating higher levels of pain catastrophizing. The levels of average pain intensity [40] and pain bothersomeness [41] in the past 7 days were assessed with a single-item measure. Pain intensity was rated on a 0 (no pain) to 10 (worst pain imaginable) scale and pain bothersomeness was rated on a 0 (not at all bothersome) to 10 (extremely bothersome) scale. The Patient Reported Outcomes Measurement Information System (PROMIS) sleep disturbance v1.0 (6a) short-form was administered to assess level of sleep difficulties in the past 7 days (mean 50, SD 10), with higher T-scores indicating greater difficulty [42]. Sleep disturbance was assessed because poor sleep is associated with worse pain [43] and opioid misuse [44,45], and because ER has been shown to improve sleep disturbance [29,30].

### Additional Efficacy Measures

The PROMIS measure short-form questionnaires were administered to assess pain-related symptoms in the past 7 days [46]. Specifically, PROMIS-pain interference v1.1 (6a) [47], physical function v2.0 (6b) [48], fatigue v1.0 (4a) [49], depression v1.0 (6a) [50], anxiety v1.0 (6a) [50], and social isolation v2.0 (4a) were used [48]. Each measure was rated on a 1‐5 scale. Responses on each measure were uploaded to the publicly available HealthMeasures Scoring Service to calculate T-scores (mean 50, SD 10), with higher scores indicating worse symptom or higher function. Additionally, current craving intensity was assessed on a 0 (not at all) to 10 (extremely) scale [51]. Finally, the 6-item Global Impression of Change (GIC) measure was administered at all post-ER class timepoints to assess the perceived improvement after the intervention. The 6 GIC items were about improvement on overall health, physical activity, social activity, work-related activity, mood, and pain, which were rated on a 7-point scale, ranging from 1 (very much worse) to 4 (no change) to 7 (very much improved) scale [52]. Adverse events were assessed at all follow-up time points.

### Preliminary Efficacy Endpoint at 1 Month

A prior online ER study (chronic pain and no medication inclusion criterion) showed significant reductions in PCS scores as early as 2 weeks post treatment. Significant reductions in pain intensity and pain bothersomeness consistently emerged at 1 month post treatment, which served as the primary efficacy endpoint [29,31,33,53]. These treatment effects were maintained at 3 months after ER class, which informed the durability timepoint for this study [29,31].

### Analysis Plan

#### Feasibility Thresholds

The following criteria were evaluated to determine feasibility. First, >75% of enrolled participants would attend ER. Second, the mean scores of the primary feasibility outcome measures would meet or exceed an 80% threshold for treatment appraisal and satisfaction (8 or higher on a 0‐10 scale) [35,54]. Content analysis was conducted by 2 authors (KE and MZ) for qualitative interview data to evaluate perceived feasibility and to identify areas for improvement.

#### Preliminary Efficacy

Repeated-measures ANOVAs were performed to assess whether pain and related outcomes significantly improved one month after ER (efficacy endpoint) and whether these improvements were maintained at 3 months (durability). The Greenhouse-Geisser correction was used to adjust the degrees of freedom when sphericity assumption was violated. When a significant main effect of time was found, false discovery rate (FDR) correction was applied to address multiplicity and risk of false positives. Cohen *d* was calculated to estimate the effect size of reduction at each time point compared to baseline, using the sample SD of mean difference adjusted by the correlation between measures.

### Power Analysis

The G*Power 3.1.9.7. software was used to calculate sample sizes needed to detect a moderate (*f*=0.25) effect in a repeated measures ANOVA, with alpha level of .05 and .30 autocorrelation between time points. The analysis indicated that the sample sizes of 36, 43, and 59 would yield the power of 90%, 95%, and 99%, respectively. The final sample of 51 corresponded to an estimated power of 98%.

### Missing Data

Of the 55 participants who attended ER, two participants withdrew consent after the ER class (3.6%) because one expressed concern about stigma related to the use of methadone or buprenorphine, and the other stated having no problem with pain catastrophizing. Two participants did not complete any follow-up surveys. Therefore, data from 51 participants who attended the class and completed at least one follow-up survey were analyzed. Missing rates ranged from 1.9% (pain intensity and pain bothersomeness at 1 mo post treatment) to 17.6% (PROMIS-measure at 2 mo post treatment). Missing values were estimated and replaced using linear regression (linear trend at point).

Multimedia Appendix 1 shows the results of repeated measures ANOVA with pairwise deleted data collected at baseline, 2 weeks post treatment, and 1 month post treatment (efficacy endpoint). Paired *t*-tests were conducted as a post hoc test to identify time points of significant symptom reduction and its maintenance up to 3 months post treatment. These results were consistent with analyses using missing data replacement.

## Results

### Participants

Table 1 summarizes the recruitment sources and demographic characteristics. Most participants were recruited from the Stanford Pain Clinic through the local research registry and study flyers (21/51, 41.2%), followed by email invitations to those who had previously participated in observational studies and agreed to be contacted for future research (14/51, 27.5%). An additional 9 of 51 participants (17.6%) self-referred after learning about the study through ClinicalTrials.gov, Stanford Pain Lab website, social media, or online searches. Notably, based on self-report, only 1 participant received opioid prescriptions from a methadone or buprenorphine clinic, whereas the majority were prescribed opioids through pain or primary clinics.

**Table 1.** Demographic characteristics (N=51)^a^.

Characteristics		Values		
Age (years), mean (SD), range		48.6 (12.2), 28-71		
Recruitment sources, n (%)				
Pain clinic–based referrals		21 (41.2)		
Previous research connections		14 (27.5)		
Online or self-referrals		9 (17.6)		
Physician referrals		2 (3.9)		
Missing		5 (9.8)		
Gender, n (%)				
Female		27 (52.9)		
Male		21 (41.2)		
Nonbinary		1 (2.0)		
Ethnicity, n (%)				
Non-Hispanic		42 (82.4)		
Hispanic		7 (13.7)		
Race, n (%)				
White or Caucasian		36 (70.6)		
Black or African American		9 (15.7)		
More than one race		4 (7.8)		
Decline to answer		1 (2.0)		
Marital status, n (%)				
Married or living together		31 (60.8)		
Divorced, separated, or widowed		11 (21.6)		
Never married		5 (9.8)		
Partnered or not living together		2 (3.9)		
Education, n (%)				
High school		3 (5.9)		
Some college, no degree		12 (23.5)		
Associate’s or vocational certificate		8 (15.7)		
Bachelor’s or higher		26 (51)		
Employment, n (%)				
Full-time		11 (21.6)		
Part-time		4 (7.8)		
Not employed		1 (2.0)		
Retired		7 (13.7)		
Not working due to medical reasons		26 (51)		
Annual income (in US$), n (%)				
<10,000		6 (11.8)		
10,000-24,999		7 (13.7)		
25,000-44,999		10 (19.6)		
45,000-64,999 (Median)		4 (7.8)		
65,000-104,999		10 (19.6)		
>105,000		9 (17.6)		
Decline to answer		3 (5.9)		
Housing status, n (%)				
Not homeless		46 (90.2)		
Homeless sheltered		2 (3.9)		
Decline to answer		1 (2)		

a Missing n=1 for race and n=2 for all the other demographic data.

Participants reported an average age of 48.6 (SD 12.2) years and were predominantly female (27/51, 52.9%), non-Hispanic (42/51, 82.4%), White or Caucasian (36/51, 70.6%). Most participants were married or were living with a partner (31/51, 60.8%). Just over half (26/51, 51%) held a bachelor’s degree or higher. While these demographic characteristics were consistent with our previous studies of participants without prescription opioids [29-31], a notable distinction was that 51% (26/51) were not working due to medical reasons, suggesting a higher burden of illness and greater disease severity than observed in prior research. The median income was between US $45,000 and US $64,999. To note, US individual annual income was $47,960 in 2022 [55]. Finally, most people were not homeless (46/51, 90.2%) and 2 of 51 (3.9) were residing in a homeless shelter.

The baseline clinical characteristics are summarized in Table 2. Most participants (42/51, 82.4%) reported experiencing pain for at least one year. While 88.2% (45/51) reported having a pain-related diagnosis, only 37 provided specific diagnoses, with an average of 2 (SD 1.6) diagnoses per participant. The five most common diagnoses were back or spine pain, arthritis, fibromyalgia, neuralgia, and migraine. Nearly half of the participants were taking methadone (24/51, 47.1%), while the remainder were on buprenorphine (27/51, 52.9%); 43.1% (22/51) met the diagnostic criteria for current OUD. The mean *DSM-5* OUD symptom severity score was 2.7 (SD 3.3), indicating overall mild OUD severity. Among the 28 participants who reported a history of mental health conditions, 26 provided specific diagnoses, with an average of 1.9 diagnoses per person (SD 0.8). Depression (18/26, 69.2%) and anxiety (13/26, 50%) were the most frequently reported diagnoses. Regarding current mental health treatment, 23.5% (12/51) were engaged in psychotherapy with a counselor, psychologist, or social worker. Additionally, 43.1% (22/51) reported prior participation in a pain management class, excluding ER.

**Table 2.** Clinical characteristics (N=51).

Characteristics		Values		
Pain duration^a^, n (%)				
<6 months		2 (3.9)		
6 and <12 months		5 (9.8)		
1 or more years (median)		42 (82.4)		
Received pain diagnosis?^a^, n (%)				
Yes		45 (88.2)		
No		4 (7.8)		
Pain diagnosis (n=37), n (%)				
Back and spinal pain condition		9 (24.3)		
Arthritis		5 (13.5)		
Fibromyalgia		5 (13.5)		
Neuralgia		5 (13.5)		
Migraine		4 (10.8)		
Connective tissue disorder		3 (8.1)		
Complex regional pain syndrome		3 (8.1)		
Stenosis		2 (5.4)		
Pelvic pain		2 (5.4)		
Orthopedic conditions		2 (5.4)		
Other^b^		9 (24.3)		
Opioid medication, n (%)				
Methadone		24 (47.1)		
Buprenorphine		27 (52.9)		
Current opioid use disorder, n (%)				
No		25 (49)		
Yes		22 (43.1)		
Mild (2-3)		10 (19.6)		
Moderate (4-5)		3 (5.9)		
Severe (6-11)		9 (17.6)		
Missing		4 (7.8)		
Ever received mental health diagnosis?^b^, n (%)				
Yes		28 (54.9)		
No		21 (41.2)		
Mental health history (n=26), n (%)				
Depression		18 (69.2)		
Anxiety		13 (50)		
Posttraumatic stress disorder		8 (30.7)		
Bipolar disorder		3 (11.5)		
Other^c^		5 (19.2)		
Currently seeing a therapist?, n (%)				
Yes		12 (23.5)		
No		15 (29.4)		
Decline to answer or missing		24 (47.1)		
Previous pain management class?^a^, n (%)				
Yes		22 (43.1)		
No		27 (52.9)		
Number of pain diagnosis (n=37), mean (SD), range		2 (1.6), 1-9		
Number of mental health diagnoses (n=26), mean (SD), range		1.9 (0.8), 1-4		

a Missing n=2.

b Other: tendonitis, pancreatitis, interstitial cystitis, restless leg syndrome, esophageal dysmotility, cerebrospinal fluid leak, viremia, stomach ulcer, frozen shoulder, sum >100% for multiple diagnoses.

c Other: attention-deficit/hyperactivity disorder, insomnia, obsessive-compulsive disorder, personality disorder, substance abuse, sum >100% for multiple diagnoses/person.

### Primary Outcome: Feasibility

The ER attendance rate of 79.7% (n=55) exceeded the preregistered attendance threshold of 75%. A total of 50 participants completed the postclass treatment appraisal and satisfaction survey. As summarized in Table 3, all item ratings exceeded the feasibility threshold of 8.0, except for concerns about privacy during the Zoom class (mean 7.3, SD 2.2); because this single item is interpreted in the reverse direction (ie, higher scores reflect greater concerns), the mean score for the privacy concern item was notably high. The mode for feasibility and acceptability ratings was 10 across all items, indicating strong endorsement. Participants gave high ratings for ER intervention (overall satisfaction, easy to understand, relevant, useful, likelihood to use the skills learned and to recommend to others, and easy to use a zoom platform: mean 8.2‐9.5). The lowest rating was feeling connected with the instructor (mean 8.1, SD 2.1).

**Table 3.** Primary outcome: ER treatment appraisal, satisfaction, and acceptability ratings (n=50).

Survey items	Values	Mode
Item response scale (0-10), mean (SD)		
Please rate your overall satisfaction with the class.	8.6 (2)	10
Was the content easy to understand?	9.5 (1)	10
How relevant was the class to you?	8.6 (2)	10
How useful was the information presented in the class?	8.2 (2.4)	10
How likely are you to use the skills and information you learned?	8.4 (2.1)	10
Rate your likelihood to recommend this class to another person who has chronic pain.	8.3 (2.4)	10
Please indicate how much you agree with the following statements		
It was easy for me to operate Zoom as a platform for attending the class.	8.8 (3.4)	10
While attending the Zoom class, I worried about privacy.	7.3 (2.2)	10
I felt comfortable engaging with the Zoom instructor and class participants.	8.4 (2.5)	10
I felt connected to the instructor.	8.1 (2.1)	10
Item responses (see below note for the three response options), n (%)		
Would you prefer to have a single class (2 hours) or two classes (1 hour each)?		
Prefer single, 2-hour class	43 (84.3)	
Prefer 1-hour two classes	5 (9.8)	
Other	2 (3.9)	

### Content Analysis of Qualitative Data

At the end of the study, participants provided open-ended feedback about the study processes in response to a set of questions related to four domains: class content, experience with the ER class and the study procedures, technical issues, and the recruitment strategies. A content analysis was conducted to code responses within these domains.

#### Class Content

All participants reported the class content easy to understand, and no participants reported discomfort with the class material. Overall, 6 participants reported that no changes were needed for future participants, while others suggested more advanced material (n=2), more time to process the class content (n=1), a more interactive component (n=1), and greater sharing of personal experiences by the moderators (n=1).

#### Experience With the ER Class and Study Procedures

Overall experiences were positive, with few concerns raised. Suggestions for improvement included incorporating more breaks (n=1) and providing more reminder calls prior to the class (n=1).

#### Technical Issues

Technical challenges were infrequently reported. Only 2 participants reported technical difficulties with Zoom and required additional support to download the relaxation audio file.

#### Recruitment Strategies

Most participants did not express concerns (n=13). One participant requested clearer expectations regarding the timing of a follow-up call after initial contact. Participants also suggested expanding advertising efforts through social media and video platforms (eg, Facebook and TikTok), printed materials on college campuses, flyers in clinic waiting rooms, direct referrals from physicians (including those in palliative care, pain management, and primary care), curated patient lists with targeted email invitations, and referrals from past participants.

In summary, the quantitative and qualitative findings indicate that the ER intervention was feasible and well accepted, as evidenced by high attendance, strong satisfaction ratings, and positive feedback regarding the class content and study procedures. Qualitative findings provided additional insight into specific areas for improvement, including participant engagement, technical support, and recruitment strategies.

### Preliminary Efficacy

**Figure 3.**
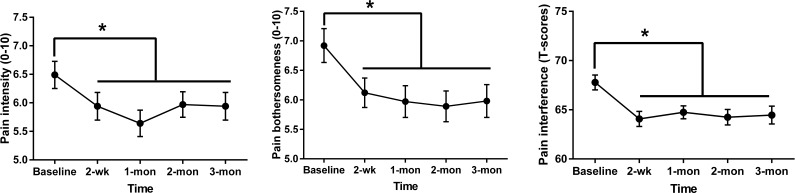
Results with false discovery rate–corrected post hoc tests revealed significant changes in average pain intensity (left), pain bothersomeness (middle), and PROMIS-Pain Interference T scores (right). * FDR corrected *P*<.05. Error bars denote SEM.

Repeated-measures ANOVAs were conducted to examine if our primary variables of interest (pain catastrophizing, average pain intensity, pain bothersomeness, and sleep disturbance) would be significantly improved after ER intervention (Table 4). The results revealed a significant main effect of time only in PCS total scores, average pain intensity, and pain bothersomeness. However, with post hoc FDR correction applied (summarized in Table 4), the PCS effect over time was lost (*P*>.07) while average pain intensity and pain bothersomeness retained significance (Figure 3). Compared to the baseline (mean 6.49, SD 1.70), pain intensity was significantly reduced at 2 weeks (*M*_DIFF_=0.55, *d*=0.41; *P*=.01) and 1 month (*M*_DIFF_=0.85, *d*=0.71; *P*<.001). Although the magnitude of the treatment effect on pain intensity was reduced to a small effect size, it remained significant at 2 months (*P*=.03; *d*=0.33) and 3 months post treatment (*P*=.05, *d*=0.28). For pain bothersomeness, compared to the baseline (mean 6.92, SD 2.05), it was significantly reduced at 2 weeks (*M*_DIFF_=0.80, *d*=0.43; *P*=.004) and 1 month post treatment (*M*_DIFF_=0.96, *d*=0.54; *P*=.001), and this reduction was maintained at 2 months (*M*_DIFF_=1.04, *d*=0.48; *P*=.002) and 3 months post treatment (*M*_DIFF_=0.94, *d*=0.44; *P*=.004).

**Table 4.** Repeated-measures ANOVA for outcome measures (N=51).

Measures	Type III (sum of squares)	*F* test (*df*^a^)	*P* value	η^b^
PCS^c^		3.01 (3.20, 160.16)	.03	0.057
Time	592.71			
Error	9837.64			
Average pain intensity		3.88 (3.03,151.28)	.01	0.072
Time	19.4			
Error	249.96			
Pain bothersomeness		4.69 (3.61, 180.62)	.002	0.086
Time	36.98			
Error	394.04			
PROMIS^d^				
Sleep disturbance		1.80 (3.22, 161.18)	.15	0.035
Time	192.45			
Error	5349.7			
Pain interference		8.61 (3.06, 153.09)	<.001	0.147
Time	485.76			
Error	2820.23			
Physical function		2.35 (3.75, 187.37)	.06	0.045
Time	63.8			
Error	1356.58			
Fatigue		1.34 (3.53, 176.60)	.26	0.026
Time	189.47			
Error	7044.62			
Depression		1.89 (3.48, 174.12)	.12	0.036
Time	211.72			
Error	5600.31			
Anxiety		0.62 (3.15, 157.45)	.61	0.012
Time	80.89			
Error	6502.87			
Social isolation		1.64 (3.11, 155.43)	.18	0.032
Time	212.23			
Error	6484.51			
Opioid craving scale (0‐10)		2.97 (2.91, 145.28)	.04	0.056
Time	22.61			
Error	381.13			

a Adjusted *df* using the Greenhouse-Geisser correction.

b Eta-squared (*η*2) of 0.01, 0.06, and 0.14 would indicate small, medium, and large, respectively.

c PCS: Pain Catastrophizing Scale.

d PROMIS: Patient Reported Outcomes Measurement Information System.

Taken together, our hypotheses were partially supported. Contrary to our hypotheses, pain catastrophizing and sleep disturbance were not significantly reduced after ER intervention, but pain intensity and pain bothersomeness were significantly reduced to a moderate degree at 1 month post treatment. While reductions in pain intensity attenuated over time, reductions in pain bothersomeness were sustained through the 3 months post treatment (durability time point).

### Additional Measures

Repeated-measures ANOVAs were performed to examine if pain interference, physical function, fatigue, depression, anxiety, social isolation, and opioid craving symptoms would be significantly improved after ER intervention (Table 4). The results revealed a significant main effect of time in PROMIS-pain interference T-scores and opioid craving. Application of FDR correction for multiplicity revealed significant changes in PROMIS-pain interference T-scores were retained (Figure 3), but not for craving (*P*>.09, Table 5). Compared to the baseline (mean 67.78, SD 5.40), pain interference T-scores were significantly reduced at 2 weeks (mean difference [*M*_DIFF_]=3.71, *d*=0.59; *P*<.001) and 1 month post treatment (*M*_DIFF_=3.04, *d*=0.61; *P*<.001) and this reduction was maintained at 2 months (*M*_DIFF_=3.54, *d*=.56; *P*<.001) and 3 months post treatment (*M*_DIFF_=3.32, *d*=.48; *P*=.001; Figure 3). Taken together, our hypotheses were partially supported. Among our additional measures, a moderate reduction was observed only in pain interference at 1 month post treatment (efficacy endpoint), and this reduction was maintained at 3 months post treatment (durability time point).

**Table 5.** Post hoc false discovery rate–corrected time-effect comparisons between the baseline and follow-ups (N=51).

Time points	Mean (SD)	M_DIFF_	SE_DIFF_	*P* value	Cohen *d*^a^
PCS^b^ total scores					
Baseline	20.18 (13.53)	Ref.^c^	Ref.	Ref.	Ref.
2 weeks	17.47 (9.72)	2.71	1.41	.08	0.27
1 month	19.42 (10.65)	0.76	1.32	.57	0.08
2 months	16.27 (9.96)	3.90	1.81	.07	0.30
3 months	16.73 (11.82)	3.45	1.60	.07	0.30
Average pain intensity					
Baseline	6.49 (1.70)	Ref.	Ref.	Ref.	Ref.
2 weeks	5.94 (1.73)	0.55	0.19	.01	0.41
1 month	5.64 (1.65)	0.85	0.17	<.001	0.71
2 months	5.97 (1.60)	0.52	0.22	.03	0.33
3 months	5.94 (1.73)	0.55	0.28	.05	0.28
Pain bothersomeness					
Baseline	6.92 (2.05)	Ref.	Ref.	Ref.	Ref.
2 weeks	6.12 (1.78)	0.8	0.26	.004	0.43
1 month	5.97 (1.92)	0.96	0.25	.001	0.54
2 months	5.89 (1.87)	1.04	0.30	.002	0.48
3 months	5.98 (1.99)	0.94	0.30	.004	0.44
PROMIS^d^-pain interference					
Baseline	67.78 (5.40)	Ref.	Ref.	Ref.	Ref.
2 weeks	64.07 (5.44)	3.71	0.88	<.001	0.59
1 month	64.74 (4.68)	3.04	0.70	<.001	0.61
2 months	64.24 (5.63)	3.54	0.88	<.001	0.56
3 months	64.46 (6.46)	3.32	0.96	.001	0.48
Craving					
Baseline	2.69 (3.48)	Ref.	Ref.	Ref.	Ref.
2 weeks	2.41 (2.90)	0.27	0.28	.45	0.14
1 month	2.51 (2.81)	0.18	0.33	.60	0.07
2 months	1.83 (2.34)	0.86	0.36	.09	0.33
3 months	2.16 (2.64)	0.52	0.32	.23	0.23

a Cohen* d* was calculated from paired-sample *t* tests.

b Pain Catastrophizing Scale

c Ref. refers to a reference time point.

d Patient Reported Outcomes Measurement Information System.

Finally, ratings on the 6-item GIC measures were analyzed to examine perceived improvement at 1 month and 3 months post treatment compared to baseline (Table 6). Mean overall improvement rating on a 1‐7 scale was modest at 1 month (mean 4.5, SD 1.1) and 3 months post treatment (mean 4.5, SD 1.2). Regarding pain improvement, 17 of 46 participants (37%) at 1 month post treatment and 21 of 49 participants (42.9%) at 3 months posttreatment rated 5 or higher on the GIC pain item.

**Table 6.** The Global Impression of Change ratings on the 6 domains.

Improvement in	1 month post treatment (n=46)			3 months post treatment (n=49)		
	Mean (SD)	Range	>4^a^ (%)	Mean (SD)	Range	>4 (%)
Overall	4.5 (1.1)	2‐6	52.2	4.5 (1.2)	2‐7	51.0
Physical activity	4.5 (0.9)	2‐6	41.3	4.4 (1.1)	2‐6	46.9
Social activity	4.3 (0.9)	2‐7	37.0	4.5 (1.2)	1‐7	38.8
Work-related activity	4.3 (0.9)	2‐7	32.6	4.3 (1.2)	2‐7	38.8
Mood	4.2 (1.1)	2‐6	43.5	4.5 (1.2)	2‐7	51.0
Pain	4.4 (0.8)	2‐6	37.0	4.2 (1.1)	2‐6	42.9

a Ratings of >4 would indicate perceived improvement after ER.

### Adverse Events Related to ER Intervention

There were no serious adverse (SAEs) and adverse events (AEs) related to the ER intervention. Regarding SAE, 1 participant reported an emergency department visit for chest and abdominal pain at 2 months post treatment. The reported AEs included (1) injury (n=9, fall, sprained ankle, neck and back injury, second-degree burn, cat bite), (2) infection (n=7, COVID-19, flu, and infection from cat bite), (3) new medical diagnosis (n=6, rheumatoid arthritis, cancer, aneurysm, rule out brain cyst, and cannabis hyperemesis syndrome), (4) medical treatment (n=6, surgery, injection, ablation, removal of 15 teeth, and low-dose naltrexone), (5) gastrointestinal issue (n=3, bowel obstruction and stomach pain), and (6) unhealed wound (n=1).

## Discussion

### Overview

This single-arm pilot study investigated online ER in individuals with chronic pain taking methadone or buprenorphine. The primary goal was to evaluate the feasibility and acceptability of the ER intervention in this population. We also examined the preliminary efficacy of ER for reducing pain catastrophizing, pain intensity, pain bothersomeness, and sleep disturbance at 1 month post treatment, as well as additional outcomes (pain interference, physical function, fatigue, depression, anxiety, social isolation, and opioid craving). Further, we tested the durability of treatment effects at 3 months post treatment.

### Feasibility and Acceptability (Primary Outcomes)

This study demonstrated acceptable feasibility of ER intervention, as indexed by a class attendance rate of 79.7% among consented participants, which exceeded the predefined threshold of 75%. Of the 55 class attenders, only 2 withdrew their consent and 2 were lost to follow-up. Additionally, the treatment engagement rate among all eligible participants was 74.3%, calculated as the proportion who attended the ER class. This rate fell within the range reported in other single-arm studies of single-session intervention for chronic pain (56% to 96%) [56-59]. Further evidence of feasibility might be the recruitment of 74 eligible participants over the course of 18 months, with 69 consenting to participate. However, the initial recruitment challenge should be noted. The original plan was to recruit participants exclusively through physician referrals from methadone or buprenorphine clinics in the United States, aiming to create a homogenous group with automatically verified use of these medications. However, relying entirely on external physician referrals resulted in zero participant recruitment for 8 months. Subsequently, we expanded recruitment using multiple methods, including physician referrals from pain and addiction clinics, self-referral via research posting on the Stanford Pain Lab website and ClinicalTrials.gov, and email invitations to patients with chronic pain through Stanford Pain Division’s research registry and prior observational studies. This shift in recruitment strategy may have influenced the composition of the study sample by preferentially enrolling individuals who were more proactive in seeking research opportunities, potentially reflecting higher health literacy or web access compared to patients recruited exclusively through physician-based referrals from methadone or buprenorphine clinics. As a result, the final sample was clinically heterogeneous with respect to OUD severity, including no OUD (49.0%), mild (19.6%), moderate (5.9%), and severe cases (17.6%). Additionally, overall OUD symptoms were mild (mean 2.7, SD 3.3). These factors should be considered when interpreting feasibility outcomes and the generalizability of the findings to broader clinical populations receiving opioid agonist therapy.

Participants reported high overall satisfaction with the class (mean 8.6, SD 2 on a 0‐10 scale), and high ratings of relevance, usefulness, and ease of understanding the material, in addition to high likelihood of using the skills and recommending the class to others (mean 8.3‐9.5). Participants also reported high ratings related to feeling connected with the instructor (mean 8.1, SD 2.1). These ratings indicate strong endorsement of web-based ER intervention. Concerns about privacy during the Zoom class were notably high, with higher scores reflecting greater privacy concerns (mean 7.3, SD 2.2). To minimize privacy concerns during the class, participants were instructed to turn off their cameras and use first names only; this method was previously used to deliver a group online program for individuals with OUD [60]. Despite this approach, one participant withdrew from the study after attending the ER class due to discomfort with the study’s focus on individuals taking methadone or buprenorphine and concerns about associated stigma. This highlights the potential impact of opioid-related stigma on engagement and retention in research and possibly group-based behavioral interventions. Accordingly, sensitive recruitment and intervention approaches should be considered. One such approach is to offer ER within the same clinics where individuals receive ongoing care for chronic pain or OUD treatment, thereby normalizing participation as part of routine care. Additionally, involvement of referring clinicians may be beneficial beyond recruitment. Initial and ongoing communication from a trusted referring physician, emphasizing ER as an evidence-based, skills-focused self-management program aimed at optimizing pain outcomes and supporting positive self-care behaviors may help reduce stigma-related concerns and reinforce the value of participation. Such clinician-supported messages may enhance engagement, retention, and acceptability of behavioral pain management interventions in this population. Although qualitative interviews were conducted to identify areas for improvement, no participants raised privacy concerns during the interviews, limiting the opportunity to explore this issue. Future study should incorporate more targeted qualitative probes, which include stratifying interviews based on reported privacy concerns in quantitative surveys, asking focused questions to better understand privacy concerns in online group interventions, and identifying strategies to address them.

### Qualitative Data

This study collected qualitative feedback regarding class content, experience with the ER class and study procedures, technical issues, and the recruitment strategies. All participants found the class content easy to understand, did not report discomfort with the class material, and had an overall positive experience with the class and study procedures. Most participants did not report technical issues with the online surveys, the Zoom, and binaural relaxation app. A few participants offered suggestions to improve the content and delivery of ER. For example, 2 participants requested more advanced content, but 1 expressed a need for additional time to process the content. This opposite request is expected for group-based intervention. One solution is to provide individualized options after the ER session. For example, offering supplemental materials for those seeking more advanced content and a brief follow-up check-in for participants who need additional process time. Delivery-related suggestions included adding interactive components and time to share personal experiences. These are expected because ER is designed as a didactic format for efficient, scalable delivery. Minor suggestions for improvement will be considered in future study designs.

### Preliminary Efficacy

Among our primary preliminary efficacy outcomes, pain intensity demonstrated moderate effects at 1 month post treatment (Cohen *d*=0.71). At 1 month, average pain intensity decreased from 6.49 (SD 1.70) at baseline to 5.64 (SD 1.65), corresponding to a statistically significant 0.85-point reduction on a 0‐10 scale. Although this moderate reduction in pain intensity reflects a positive response trajectory, it falls below thresholds for clinical meaningful change (eg, ≥2-point or 30% reduction) [61,62]. These effects attenuated over time, with smaller but significant reductions observed at 2 months (mean 5.97, SD 1.60; *Δ*=0.52; Cohen *d*=0.33) and 3 months (mean 5.94, SD 1.73; *Δ*=0.55; Cohen *d*=0.28). FDR correction increases confidence that these findings represent true effects rather than spurious findings.

Pain bothersomeness showed a similar but slightly more sustained pattern of improvement. At 1 month post treatment, pain bothersomeness decreased from 6.92 (SD 2.05) at baseline to 5.97 (SD 1.92), representing a statistically significant 0.95-point reduction on a 0‐10 scale (Cohen *d*=0.54). Reductions were maintained at 2 months (mean 5.89, SD 1.87; *Δ*=1.03; Cohen *d*=0.48) and 3 months (*M*mean 5.98, SD 1.99; *Δ*=0.94; Cohen *d*=0.44). Despite these consistent and statistically significant improvements, thresholds for clinically meaningful change in pain bothersomeness have not yet been established. As such, while ER may reduce the subjective burden of pain, its clinical impact on pain bothersomeness may be modest as currently delivered.

For the other efficacy outcome, pain interference also demonstrated moderate effects across follow-up time points. At 1 month post treatment, PROMIS pain interference T-scores decreased from 67.78 (SD 5.40) at baseline to 64.74 (SD 4.68), corresponding to a statistically significant 3.04-point reduction (Cohen *d*=0.61). These effects were maintained at 2 months (mean 64.24, SD 5.63; *Δ*=3.54; Cohen *d*=0.56) and 3 months (mean 64.46, SD 6.46; *Δ*=3.32; Cohen *d*=0.48). However, these reductions were less than 5 points, suggesting that these changes likewise fall below the thresholds for clinically meaningful improvement [63].

In summary, this pilot study suggests that ER, as a single-session intervention, may yield its statistically significant and durable effects on pain-related outcomes up to 3 months. However, modification of the intervention may be necessary to enhance its clinical impact in this population. As this was the first test of ER in this population, our hypotheses were based on prior studies conducted in other chronic pain populations. Notably, the current study did not demonstrate reductions in pain catastrophizing. While this contrasts with many ER trials conducted in chronic pain samples without opioid-related inclusion criteria, it is consistent with a growing subset of ER studies conducted in populations taking prescription opioids or with opioid misuse. In the current sample of the patient population taking methadone or buprenorphine, 43.1% (22/51) met criteria for current OUD, suggesting that ER’s effect on cognitive-affective pain processes may differ in opioid-exposed population. These findings highlight the need for future research to refine ER to optimize treatment outcomes as well as improve pain-related coping in this patient population.

In multiple other studies, ER was found to significantly and clinically meaningfully reduce pain catastrophizing, with this variable often showing the largest treatment effects and multidimensional benefits. Indeed, randomized controlled trials (RCTs) of ER in general chronic pain have revealed that ER was superior to control and significantly improved multiple variables, including pain catastrophizing, pain intensity, pain bothersomeness, sleep disturbance, pain interference, physical function, fatigue, depression, anxiety, and social isolation. Specifically, one 3-arm RCT in 263 patients with chronic low back pain showed that, compared to the health education control, in-person ER was significantly better in improving all secondary (Glass *d*s=0.54‐0.93^30^) and tertiary outcome measures (Glass *d*s=0.31‐0.45^30^) at 3 months. Another RCT in a general chronic pain sample (n=104) showed that, compared to a waitlist control, online ER was significantly better in improving all secondary outcome measures (Cohen *d*s=0.60‐0.67) at 3 months post treatment and sleep disturbance was not measured [31]. This RCT also showed that online ER was significantly better in improving some of the tertiary outcome measures at 3 months post treatment such as anxiety and anger (Cohen *d*s=0.68 and 0.59, respectively), but no significant between-group differences were found for pain interference, physical function, and social isolation at 3 months [31]. These two RCTs suggest that regardless of delivery method (in-person or online), ER significantly improved pain catastrophizing, pain intensity, and pain bothersomeness to a moderate to large degree, but pain interference was moderately improved only after in-person ER (Glass *d*=0.64) [29,30].

The surprising lack of effects for pain catastrophizing and other variables in the current trial may relate to OUD, prescription opioids, or both. To date, among 13 completed ER clinical trials [29-36,53,54,64-66], 8 show strong effects for reducing pain catastrophizing, with all of those being chronic pain studies with no enrollment criteria for prescription opioids [29-33,53,54,65]. In contrast, the 5 other studies were conducted in patients taking prescription opioids, either for chronic pain or post surgery; while we found clinical benefits resulting from ER for certain outcomes (eg, opioid reduction and pain reduction), we found null effects for pain catastrophizing reductions in these opioid-taking populations [34,36,64-66]. A single-arm study examined the feasibility and preliminary efficacy of online ER in 41 individuals prescribed daily opioids (≥10 morphine-equivalent daily dose), including both short- and long-acting medications [35]. That study demonstrated a significant small improvement in pain catastrophizing, pain intensity, and pain interference at 3 months post treatment (Cohen *d*s=0.27‐0.39). Ziadni et al (in review; protocol [65]) conducted a RCT of ER in 213 patients taking daily prescription opioids and found no effects for PCS reduction; this study was a notable standout for showing minimal clinical benefits overall for ER. Another recently completed RCT of a digital (asynchronous) version of ER in 231 individuals with prescription opioid misuse (manuscript in development; protocol [66]) yielded significant ER impacts on pain and opioid outcomes but null effects for pain catastrophizing. Two ER RCTs in patients who underwent surgery similarly found no effect for pain catastrophizing but significant effects for reducing opioid use and pain after surgery [34,64], although a floor effect for pain catastrophizing at baseline is likely a factor. For this study, baseline pain catastrophizing was substantial and thus eliminating a floor effect hypothesis. However, the findings for these 5 studies align with a broader understanding that central nervous system calming imparts salutary effects even in the absence of cognitive and emotional distress. Moreover, population-specific differences in ER’s mechanisms of action may exist. Future studies should evaluate multiple pain-coping mechanisms to elucidate ER’s mechanisms of action in this population.

Finally, it is unclear whether additional treatment “dosing” might yield effects on pain catastrophizing and other variables where no effects were observed (eg, opioid craving). For instance, booster sessions, intensive or multisession interventions, or a different type of treatment may be needed to reduce pain catastrophizing and further optimize outcomes in this population. Multisession, behavioral interventions for chronic pain have shown slightly different patterns, demonstrating consistent efficacy in opioid-related outcomes, but limited effect on pain-related outcomes for this population. Specifically, multisession CBT and MORE consistently demonstrated reductions in opioid misuse, opioid craving, and other substance use [21,23-25], but their effects on pain-related outcomes were predominantly non-significant [21-25]. This contrasting pattern may be attributable to differences in sample characteristics related to OUD and pain status. The CBT and MORE studies recruited participants taking methadone for OUD [21,24], whereas the current study recruited participants taking methadone or buprenorphine for either chronic pain or OUD. As a result, 52.9% (27/51) of our participants were taking buprenorphine, and 43.1% (22/51) had a current OUD diagnosis. Craving levels in our sample were low at baseline (mean 2.69, SD 3.48 on a 0‐10 scale) and remained low throughout the 3-month follow-up period (mean 1.83, SD 2.34 to 2.51, SD 2.81), in contrast to its levels in the MORE study sample at baseline (mean 4.5 on a 0‐10 scale). This floor effect may have limited this study’s ability to detect reductions in craving following ER. The low craving observed in our sample, drawn from pain clinics and self-referral, may reflect a relatively stable group whose opioid craving was already well managed with opioid agonist or partial-agonist treatment. Conversely, baseline pain levels were higher in our sample (mean 6.5, SD 1.7) compared to those in the CBT (mean 5.9, SD 1.5) [21] and MORE (mean 4.7, SD 3.1) [24] studies. These differences in OUD and pain characteristics should be considered when comparing treatment outcomes. Additionally, treatment hours should be taken into account: the current outcomes are based on a single-session intervention (2 h), whereas other outcomes are based on 12-session CBT (6‐9 h) and 8-session MORE (16 h).

### Limitations

Our findings should be interpreted considering study limitations. First, this single-arm pilot trial was primarily designed to examine the feasibility and acceptability of ER in individuals with chronic pain who were taking methadone or buprenorphine. Therefore, causal inferences about ER effect cannot be assumed. Observed improvements in this study could reflect regression to the mean, natural symptom variability, or nonspecific factors such as participant expectancy. Nevertheless, prior RCTs in general chronic pain populations (not specific to opioid use) have shown that ER produces clinically meaningful improvements in pain-related outcomes. Additional study, particularly an RCT of ER, is needed to establish its efficacy and exclude alternative explanations in this population. Second, although our sample size was sufficient to detect moderate changes over time, this study was likely underpowered to detect small effects, as the a priori power analysis assumed a moderate treatment effect. Several outcomes in the current study were small-to-moderate effect sizes, which may have limited power to detect smaller effects reliably. To address this limitation, we applied less conservative FDR correction to balance control of type I and type II errors. Our outcomes remained statistically significant after FDR correction, but null findings should be interpreted with caution given the limited power of this pilot study. Third, missing data were addressed using two approaches, regression-based imputation (linear trend at point) and pairwise deletion (see Multimedia Appendix 2). Consistent results across these two methods indicate that our results were reliable regardless of the methods used to address missing data. Fourth, the study sample consisted of predominantly white, non-Hispanic or Latino, educated individuals, with high rates of unemployment, which limits study generalizability. Future trials should examine ER efficacy in diverse and underserved patient populations who often experience greater barriers to treatment [67]. Lastly, the final sample was not representative of individuals receiving OUD treatment, but instead primarily comprised individuals with chronic pain who were prescribed methadone or buprenorphine. During the first 8 months of the study, recruitment efforts targeting specialty OUD treatment clinics were unsuccessful mainly because this was an unfunded pilot supported by a small amount of internal resources. Due to financial constraints, we were an external study team seeking to post study recruitment flyers in various clinics with no local clinic coinvestigators. Based on low engagement, we expanded our study recruitment to include pain clinics and self-referrals, where the majority of the study sample was derived. As such, the current findings do not generalize to OUD treatment environments, and future research is needed in this population specifically. Future research is also needed in other clinic environments, such as methadone clinics and pain clinics, with study designs that engage clinicians as coinvestigators to enhance local patient recruitment. Our recruitment challenges underscore the need to have pain treatment studies embedded into the clinic environment (ie, as a recruitment or study site) with local study leadership oversight and full participation from clinic staff.

### Conclusions

Our findings suggest that online ER appears feasible and is a promising intervention that may improve pain-related outcomes in individuals with chronic pain taking methadone and buprenorphine. Disseminating brief, accessible, and scalable pain psychology interventions, such as ER, may enhance more equal access to effective behavioral pain treatments and reduce pain burden. A fully powered RCT in diverse patient participants is needed to determine ER efficacy in patients who are receiving methadone or buprenorphine for chronic pain or OUD.

## Supplementary material

10.2196/86070 Multimedia Appendix 1 Repeated measures ANOVA for outcome measures up to the primary study endpoint (1 month).

10.2196/86070 Multimedia Appendix 2 Post hoc pairwise *t* test to compare outcomes at the baseline and follow-up points.

## Abbreviations

AE: adverse event
CBT: cognitive behavioral therapy
*DSM-5*: *Diagnostic and Statistical Manual of Mental Disorders* (Fifth Edition)
ER: Empowered Relief
FDR: false discovery rate
GIC: Global Impression of Change
HIPAA: Health Insurance Portability and Accountability Act
MORE: Mindfulness-Oriented Recovery Enhancement
OUD: opioid use disorder
PCS: Pain Catastrophizing Scale
PROMIS: Patient Reported Outcomes Measurement Information System
RCT: randomized controlled trial
REDCap: Research Electronic Data Capture
SAE: serious adverse event
