# Supplementary material for: Feasibility and Preliminary Efficacy of Empowered Relief in Patients With Chronic Pain Taking Methadone or Buprenorphine: Single-Arm National Pilot Study
Source: JMIR Form Res. 2026 Mar 11;10:e86070. doi: 10.2196/86070 (PMC12978655; doi:10.2196/86070)
Supplement: Multimedia Appendix 1 [file formative-v10-e86070-s001.docx]

Appendix 1.1 Repeated measures ANOVA for outcome measures up to the primary study endpoint (1-month)

| Measures | | n |  | Type III Sum of Squares | *df** | *F* | *p* | *η*^2^ |
| --- | --- | --- | --- | --- | --- | --- | --- | --- |
| PCS | | 43 | Time | 126.62 | 1.5 | 2.30 | .121 | .052 |
|  |  |  | Error | 2312.71 | 64.5 |  |  |  |
| Average pain intensity | | 46 | Time | 14.97 | 2.0 | 9.14 | < .001 | .169 |
|  | |  | Error | 73.70 | 89.3 |  |  |  |
| Pain bothersomeness | | 46 | Time | 18.36 | 1.9 | 6.06 | .004 | .119 |
|  | |  | Error | 136.30 | 87.0 |  |  |  |
| PROMIS | Sleep disturbance | 43 | Time | 37.04 | 1.8 | 1.21 | .301 | .028 |
|  |  |  | Error | 1283.73 | 76.6 |  |  |  |
|  | Pain interference | 43 | Time | 262.58 | 1.7 | 10.24 | < .001 | .196 |
|  |  |  | Error | 1077.40 | 70.7 |  |  |  |
|  | Physical function | 43 | Time | 14.40 | 1.9 | 1.32 | .271 | .031 |
|  |  |  | Error | 456.75 | 81.1 |  |  |  |
|  | Fatigue | 43 | Time | 32.18 | 1.7 | 0.73 | .468 | .017 |
|  |  |  | Error | 1854.19 | 73.1 |  |  |  |
|  | Depression | 43 | Time | 79.50 | 1.6 | 1.94 | .159 | .044 |
|  |  |  | Error | 1719.82 | 68.3 |  |  |  |
|  | Anxiety | 43 | Time | 32.34 | 1.8 | 0.68 | .493 | .016 |
|  |  |  | Error | 1997.04 | 74.7 |  |  |  |
|  | Social Isolation | 43 | Time | 27.21 | 1.9 | 0.59 | .545 | .014 |
|  |  |  | Error | 1926.00 | 79.0 |  |  |  |
| Opioid craving scale | | 43 | Time | 2.67 | 1.5 | 0.86 | .396 | .020 |
|  |  |  | Error | 130.00 | 61.3 |  |  |  |

Note: *adjusted *df* using the Greenhouse-Geisser correction
