# Supplementary material for: Feasibility and Preliminary Efficacy of Empowered Relief in Patients With Chronic Pain Taking Methadone or Buprenorphine: Single-Arm National Pilot Study
Source: JMIR Form Res. 2026 Mar 11;10:e86070. doi: 10.2196/86070 (PMC12978655; doi:10.2196/86070)
Supplement: Multimedia Appendix 2 [file formative-v10-e86070-s002.docx]

Appendix 1.2 Post-hoc Pairwise t-test to compare outcomes at the baseline and follow-up points

|  | Baseline | | Post-Treatment | | | |  |  |  |  |
| --- | --- | --- | --- | --- | --- | --- | --- | --- | --- | --- |
|  | M | SD |  | n | M | SD | M_DIFF_ | SE_DIFF_ | *p* | *d* |
| Average pain intensity | 6.4 | 1.7 | 2-week | 46 | 5.9 | 1.8 | 0.46 | 0.19 | .023 | 0.26 |
|  | 6.5 | 1.7 | 1-month | 49 | 5.6 | 1.4 | 0.86 | 0.17 | < .001 | 0.51 |
|  | 6.4 | 1.7 | 2-month | 42 | 6.0 | 1.8 | 0.48 | 0.24 | .058 | 0.28 |
|  | 6.5 | 1.7 | 3-month | 49 | 5.9 | 1.7 | 0.57 | 0.28 | .050 | 0.33 |
| Pain bothersomeness | 6.8 | 2.1 | 2-week | 46 | 6.1 | 1.9 | 0.74 | 0.28 | .011 | 0.37 |
|  | 7.0 | 2.1 | 1-month | 49 | 6.0 | 2.0 | 1.00 | 0.26 | < .001 | 0.50 |
|  | 6.9 | 2.1 | 2-month | 42 | 5.9 | 2.1 | 1.00 | 0.34 | .006 | 0.48 |
|  | 6.9 | 2.1 | 3-month | 49 | 6.0 | 2.0 | 0.96 | 0.31 | .003 | 0.47 |
| PROMIS-pain interference | 67.9 | 5.7 | 2-week | 46 | 64.1 | 5.7 | 3.84 | 0.97 | < .001 | 0.68 |
|  | 67.5 | 5.4 | 1-month | 46 | 64.7 | 4.9 | 2.82 | 0.72 | < .001 | 0.55 |
|  | 67.5 | 5.7 | 2-month | 42 | 34.3 | 6.2 | 3.17 | 1.03 | .004 | 0.53 |
|  | 67.8 | 5.5 | 3-month | 49 | 64.5 | 6.6 | 3.29 | 0.99 | .002 | 0.54 |
